# Ex vivo biophysical characterization of a hydrogel-based artificial vitreous substitute

**DOI:** 10.1371/journal.pone.0209217

**Published:** 2019-01-07

**Authors:** Kai Januschowski, Sven Schnichels, José Hurst, Christine Hohenadl, Charlotte Reither, Annekatrin Rickmann, Lisa Pohl, Karl-Ulrich Bartz-Schmidt, Martin S. Spitzer

**Affiliations:** 1 Eye Clinic Sulzbach-Saar, Sulzbach-Saar, Germany; 2 University Eye Clinic Tübingen, Centre for Ophthalmology, Tübingen, Germany; 3 Croma Pharma GmbH, Leobendorf, Austria; 4 University Eye Clinic Hamburg-Eppendorf, Hamburg, Germany; University of Cologne, GERMANY

## Abstract

**Purpose:**

To characterize the biophysical properties of an artificial vitreous body substitute (VBS), which consists of a biocompatible, cross-linked, hyaluronic acid (HA)-based hydrogel, by analysing the VBS’s influence on intraocular pressure (IOP) and retinal integrity in distinct ex vivo eye models in order to evaluate the its potential for in vivo biocompatibility testing.

**Methods:**

Pig eyes were obtained immediately postmortem, and VBS was injected after core-vitrectomy. IOP was followed for 24 h (n = 5). VBS influence on retinal integrity was investigated using isolated bovine retinas superfused with an oxygen saturated nutrient solution. An electroretinogram (ERG) was recorded on explanted bovine retinae using silver/silver chloride electrodes; after application of VBS for 2 min, a washout period of 70 min was employed. The percentage of a-and b-wave reduction at the end of the washout phase was compared to baseline values (n = 5). Data were calculated throughout as the mean and the standard deviation. qRT-PCR (Bax/Bcl–2-ratio, GFAP- and PGP9.5-levels) or western blot analysis was used to test for toxicity of Princess Volume after 24 h (and β-3 tubulin with GAPDH as a control gene). Significance was estimated by Student´s t-test; p ≤0.05 was considered to be statistically significant.

**Results:**

The IOP increased non-significantly by 10% after 24 h. Short-term biocompatibility testing using isolated superfused bovine retinas showed neither significant reductions of the b-wave nor the a-wave amplitudes (b-wave reduction 14.2%, p>0.05; a-wave reduction 23.9%, p>0.05). qRT-PCR and western blot analysis did not reveal significant toxicity after 24 h.

**Conclusions:**

The manufactured HA-based hydrogel showed highly favourable biophysical characteristics in the explored ex vivo models, justifying in vivo studies enabling the assessment of biocompatibility.

## Introduction

Vitreous tamponades are used for the treatment of complicated cases of retinal detachment. The most common tamponades include expansive gases and silicone oil, but their physicochemical characteristics and associated side effects limit their clinical application [1]. Gas has the advantage that it slowly diffuses out of the vitreous cavity. However, as long as the eye is gas-filled, the patient is markedly impaired due to the differing refractive index. Many complicated retinal pathologies require silicone filling. Silicone oils are not biodegradable, requiring a second surgery to remove the tamponade. Refractive changes and the multitude of risks associated with a second surgery, including the development of secondary glaucoma, have triggered a search for new tamponades with properties more similar to the human vitreous, a viscoelastic gel consisting of 98% water and a matrix of hyaluronic acid and collagen [2, 3].

An ideal vitreous tamponade should be injectable, transparent, biocompatible, stable for a certain period of time and fully biodegradable. It should be hydrophilic with a comparable refractive index (close to 1.33) but a higher viscosity and elasticity similar to that of the human vitreous body [2]. This property is crucial because compared to hydrophobic gases and silicon oils, hydrophilic gels have less surface tension and no buoyancy. Therefore, a higher viscosity and a volume increase due to controlled swelling should instead support reattachment and stabilization of the retina [4]. It seemed natural to develop hydrogels based on hyaluronic acid (HA), since these gels could potentially meet those criteria [5, 6].

HA is a glycosaminoglycan co-polymer of D-glucuronic acid and *N*-acetyl-D-glucosamine connected through alternating ß-1,4 and ß-1,3 glycosidic bonds. The axial hydrogen atoms form a nonpolar, hydrophilic face, thereby creating a twisting ribbon structure. In physiological solutions, a hyaluronan molecule imitates the form of an expanded random coil structure that occupies a very large domain; therefore, HA forms a viscous compound [5, 6].

Polymers, including HA, have been heavily investigated in the last several decades, but to date, none has fully met expectations either due to a short intravitreal half-life, inflammatory or even toxic effects at the retina and in the vitreous cavity, or little efficacy in the treatment of retinal detachments [4, 7].

Thus, early clinical studies by Pruett *et al*. with sodium hyaluronate (HYVISC) resulted in a reattached retina in only 18% of the treated patients compared to 70–90% treated with silicone oil or gas tamponades. The short residence time of less than 14 days of the hydrogel in the vitreous cavity was thought to account for the unsatisfying results. In addition, an increase in IOP was observed as an unwanted side effect [8]. These findings demonstrated that the native form of HA is not suitable because it degrades rapidly and may, due to its pronounced intrinsic swelling capacities, lead to an increase in IOP.

To overcome the problem of rapid biodegradation of native HA, more recent approaches considered the use of cross-linked polymer gels [9, 10]. Cross-linking was achieved by means of dihydrazides, chemical cross-linkers that create stable bonds between the HA molecules via carboxyl groups or by photocrosslinking of modified glycidyl methacrylate-HA conjugates with *N*-vinyl-pyrrolidinone and UV-light. In particular, the UV-cross-linked HA hydrogel revealed excellent biophysical characteristics, a refractive index of 1.338 and good biocompatibility. Moreover, in an in vivo rabbit model, it was demonstrated that the cross-linked hydrogels remained inside the eye for at least 6 months. No inflammatory or toxic reactions and no lens opacifications were observed [9, 10].

In this study, a vitreous substitute based on cross-linked thiolated HA was evaluated in order to determine whether in vivo biocompatibility testing could be performed. The thiol-modified polymer is able to build stable hydrogels by natural formation of disulfide bridges and thus does not require the addition of chemical cross-linkers or other manipulation. The generated hydrogel was characterized with respect to optical and rheological properties and subsequently tested as a vitreous substitute in two distinct ex vivo eye models in order to investigate short-term biocompatibility (in respect to ERG changes) and IOP development.

## Materials and methods

### Hydrogel preparation

For generation of an artificial VBS, a viscoelastic hydrogel was prepared from chemically derivatized thiol-substituted hyaluronic acid. The test item was a biodegradable, clear, viscous hydrogel, free of visible particles, homogenized and prepared as an injectable gel implant, which had been steam-sterilized. Formulations were prepared in physiological phosphate buffer (290 mOsmol/kg, pH 7.4) that contained 1% HA. Crosslinking was achieved by inducing disulfide bridge formation in the substituted thiol groups [11].

### Rheological analysis

A MCR101 rheometer equipped with a 50 mm cone-plate (CP50-1) measuring device (Anton Paar, Graz, Austria) was used for rotational and oscillatory rheological analysis. The linear viscoelastic range was determined by amplitude sweep measurements for each sample separately and the determined deformation γ was adjusted accordingly for the following frequency sweep measurements (n = 5). Storage (G’) and loss modulus (G”) were determined and the ratio of viscous and elastic parts (Tan δ) were calculated accordingly using the RheoPlus software (Anton Paar, Graz, Austria) [11].

### Determination of refractive index

Before measurement, sample and device (Krüss refractometer AR 2008) were adjusted to 35°C ± 2°C. Double distilled water was measured as a control and the internal standard value adjusted accordingly. Approximately 1 g of hydrogel was applied to the prism and the refractive index (RI) was determined at 589 nm (n = 5). In compliance with ISO16672:2003, RI was also determined at 546 ± 10 nm using a Carl Zeiss refractometer (CZ11401) and an LED lamp emitting at 546 nm.

### Determination of injection force

The injection force of the manufactured hydrogel (5 ml glass syringe) was determined with a motorized device (Mecmesin, West Sussex, UK) by pressing the substance (12 mm/min) through a 20 G cannula (Geuder 33051). Measurements were performed in triplicate, and the respective mean value is reported as the result.

### Modified porcine anterior chamber model

A variation of the porcine anterior chamber model previously described by Johnson et al. [12, 13] was used in the present study. The main difference is that the lens was not removed in this approach. Pig eyes were obtained from a local abattoir (Slaughterhouse eG Böblingen, Riedbrunnenstraße 5, 71116 Gärtringen, Germany) immediately after the animal’s death. All experiments were performed under sterile conditions; 5% povidone/iodine dilution was applied to the enucleated eye. The method was previously described more detailed [14], but in brief, a pars plana vitrectomy (ppV) was performed in a pig eye under a standard ophthalmic operating microscope (Carl Zeiss Meditec, Inc., Oberkochen, Germany) by two surgeons. A standard 23-gauge vitrectomy system was used (PentaSys 2, Fritz Ruck Ophthalmologische Systeme GmbH, Eschweiler, Germany); however, injection of VBS was performed using a 20-gauge Weber-needle. Initially, a sclerotomy was placed in the infratemporal quadrant, and an infusion line was connected and sutured with 7–0 vicryl (Johnson & Johnson Intl, New Brunswick, NJ, USA). Two further sclerotomies were similarly placed through the pars plana in the two superior quadrants, and a light source and vitrector were inserted into the vitreous. After core vitrectomy, a posterior vitreous detachment was created followed by careful vitrectomy of the peripheral vitreous base. Afterwards, fluid-air exchange was performed, and the VBS was injected via a 20-gauge Weber-needle until egress through the second incision was witnessed. At the end of the procedure, the infusion cannula was removed, and the incisions were sutured. No leaks were witnessed. The whole eye was fixed on a supporting device and cannulated with a 27-gauge catheter. The catheter was placed between the anterior plane of the iris and the inner surface of the cornea and connected to a microsyringe pump. The whole system was perfused with DMEM supplemented with 1.5 mg/ml glucose, 1% (vol/vol) foetal calf serum (FCS) and antibiotics (100 U/ml penicillin, 0.1 mg/ml streptomycin, and 17 g/ml gentamycin; all from Invitrogen, Germany) at a constant flow rate of 4.5 μL/min. The isolated eyes were maintained in an incubator (Biocenter 2001, Salvis-Lab, Renggli AG, Switzerland) at 37°C, while the IOP was continuously monitored with 142PC01G pressure sensors (Honeywell Sensing and Control Minnesota, USA). Data were secured by the data logger AD 128 (Valitec, Value in Technology Inc., Ohio, USA) and processed using the software DataReady (Data Ready Technologies, Florida, USA). The IOP determined after 24 h was compared to the average pressure (mean values) measured directly after the surgery.

### qRT-PCR and western blot analysis

Half of a porcine eyecup was used to prepare tissue punches after 24 h of exposition (Princess Volume, PV). These samples were then used either for qRT-PCR or western blot analysis. Expression levels of Bax/Bcl–2, GFAP and PGP9.5 were analysed by qRT-PCR (n = 5). The amount of β-3 tubulin (n = 4) was assessed via western blot analysis. GAPDH was used as a control gene. Student’s t-test was used for statistical analysis (p<0.05). The standard deviation of the mean was calculated.

### Superfused bovine retina model

Bovine eyes were obtained directly postmortem ((Slaughterhouse eG Böblingen, Riedbrunnenstraße 5, 71116 Gärtringen, Germany) and transported in a serum-free standard medium containing 120 mM NaCl, 2 mM KCl, 0.1 mM MgCl_2_, 0.15 mM CaCl_2_, 1.5 mM NaH_2_PO_4_, 13.5 mM Na_2_HPO_4_ and 5 mM glucose with a final pH of 7.8, protected from light. The preparation was performed as described earlier [15]. The electroretinogram (ERG) was recorded in the surrounding nutrient medium *via* two silver/silver-chloride electrodes placed on either side of the retina. After recording, the chamber containing a piece of retina was placed in an electrically and optically insulated box. A roller pump kept the superperfusion velocity at 1 ml/min, while the temperature was kept constant at 30°C. The superperfusion medium was pre-equilibrated and saturated with oxygen. After the retina was dark-adapted for 1 h at 30°C under constant superperfusion, the ERG was elicited at intervals of 5 min using a single white xenon flash for stimulation. A flash intensity of 6.3 mlx at the retinal surface was set using calibrated neutral density filters (Kodak Wratten Filter). Ten-microsecond durations of light stimulation were controlled with a timer (Photopic Stimulator PS33 Plus; Grass, Warwick, RI). The ERG was filtered and amplified (100 Hz high filter, 50 Hz notch filter, 100000× amplification) using a Grass RPS312RM amplifier. With an analog-to-digital data acquisition board (PCI-MIO-16XE-50; National Instruments, Austin, Texas), the data were processed and transferred to a desktop computer (PC compatible).

The retina was superfused with serum-free standard medium and stimulated repeatedly until stable b-wave amplitudes were recorded. Stable responses were achieved after approximately 2 h of superperfusion. VBS was applied epiretinally, while superperfusion was stopped for 2 min. Subsequently, VBS was removed, superperfusion was restarted, and ERG recording continued for an additional 5 min. The superperfusion with standard solution was resumed for another 70 min to monitor b-wave recovery. The b-wave amplitude was measured from the trough of the a-wave to the peak of the b-wave [16].

To investigate the effect on the photoreceptor potential, the b-wave was suppressed by adding 1 mM aspartate to the nutrient solution before the actual testing started. Under these conditions, the influence of VBS on the photoreceptors was analysed. After recording a stable photoreceptor potential for 30 min, we proceeded as described above. A series of 5 independent experiments with different retinas was conducted. The recovery of a- and b- waves was compared to the corresponding amplitudes after application of VBS and at the end of the washout [16].

### Data analysis

For statistical analysis, JMP statistical software (version 11.0, SAS Institute Inc., Cary, NC, USA) was used. Data were calculated throughout as the mean and the standard deviation. Significance was estimated by Student´s t-test; p ≤0.05 was considered to be statistically significant.

## Results

### Physicochemical characteristics of disulfide cross-linked HA hydrogel

**Fig 1:**
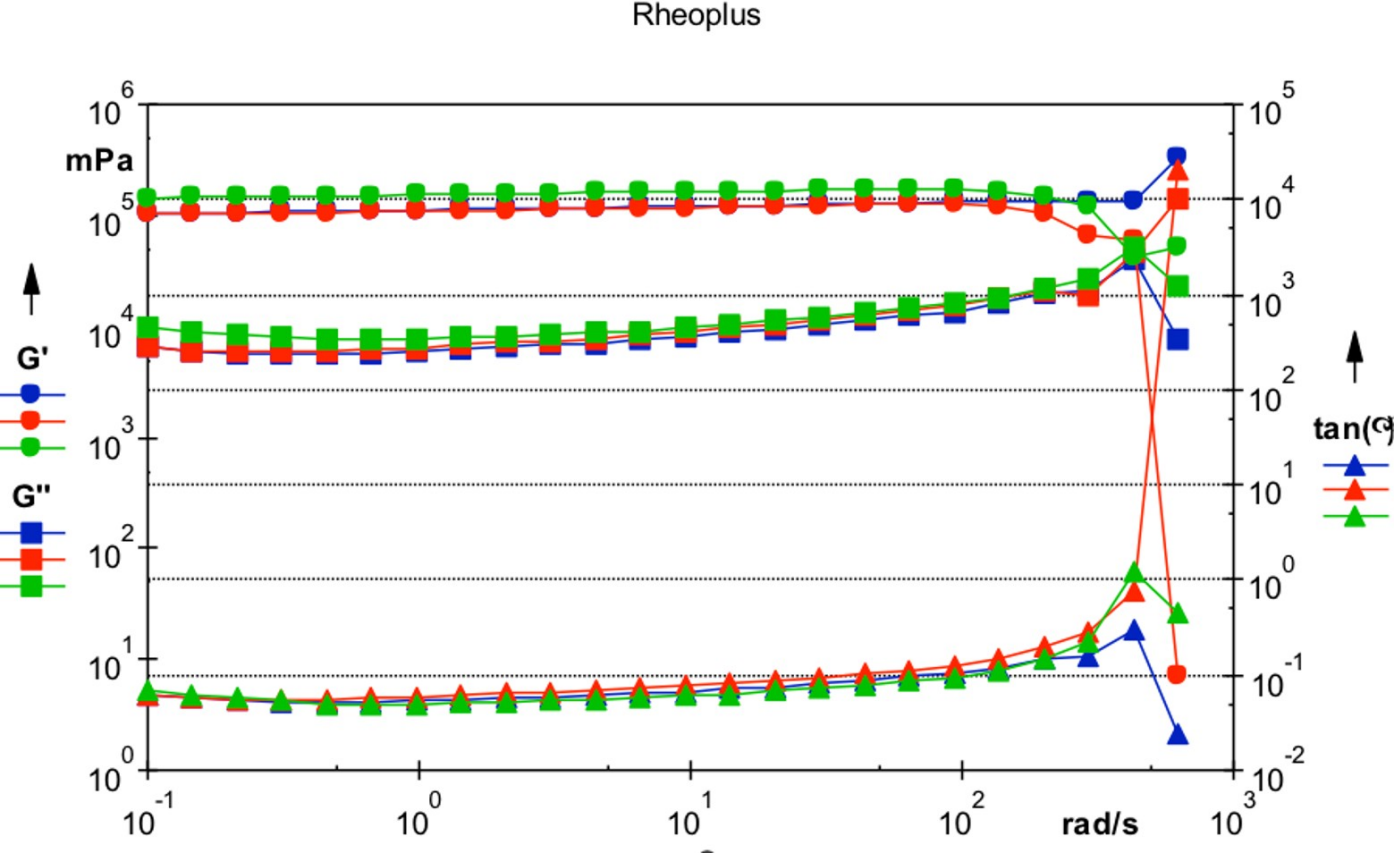
Overview of physicochemical characteristics of VBS. The blue, red and green points represent the storage moduli, the cubes represent the loss moduli (20-gauge needle) and the triangles the elasticity.

For generation of an artificial VBS, a viscoelastic hydrogel (1% HA in 100 mM physiological phosphate buffer) with thiolated HA was prepared by induction of disulfide bridge formation. The generated VBS revealed a refractive index of 1.34 at 589 nm and 1.32 at 546 nm, respectively. Rheological analysis with hydrogel pre-sheared by ejection from a 5 ml syringe through a 20-gauge needle resulted in a storage modulus (G’; ω = 1 1/s) of 128972 mPa and a loss modulus (G”; ω = 1 1/s) of 5750 mPa, indicating the highly elastic nature of the generated VBS. This is also expressed by the Tan δ, which was accordingly calculated as 0.045 (Fig 1). As a third parameter determining the applicability of the generated hydrogel as VBS, the injection force was measured. Again, a 20-gauge cannula was used during the experiments, reflecting the common situation in human vitrectomies. Under the selected conditions, an injection force of 7.5 N was measured, which is comparable to the force required to inject ophthalmic viscoelastic devices (OVDs) during cataract surgery.

In conclusion, the newly generated HA-based hydrogel revealed all basic physical characteristics required for its application as a vitreous substitute (Table 1). Consequently, the hydrogel was evaluated in a next step for its effect on intraocular pressure using a porcine ex vivo anterior chamber model.

**Table 1:** Physicochemical characteristics of the test item (1% thiol-substituted HA, in physiological phosphate buffer, naturally crosslinked). Storage (G’) and loss modulus (G”) are determined at ω = 1 1/s and the ratio of viscous and elastic parts are calculated (Tan δ). Complex viscosity was also determined at ω = 1 rad/sec.

Refractive index 589/546 nm	pH	Osmolality mOsmol/kg	Injection force (20 G needle)	Rheology (20G/23G)			Complex viscosity η* Pa.s
				G‘ mPa	G‘‘ mPa	TanΔ (G‘‘/G‘)	
1.34/1.32	7.4	290	7.5 N	128972	5750	0.045	148

### Evaluation of VBS effects on IOP

**Fig 2:**
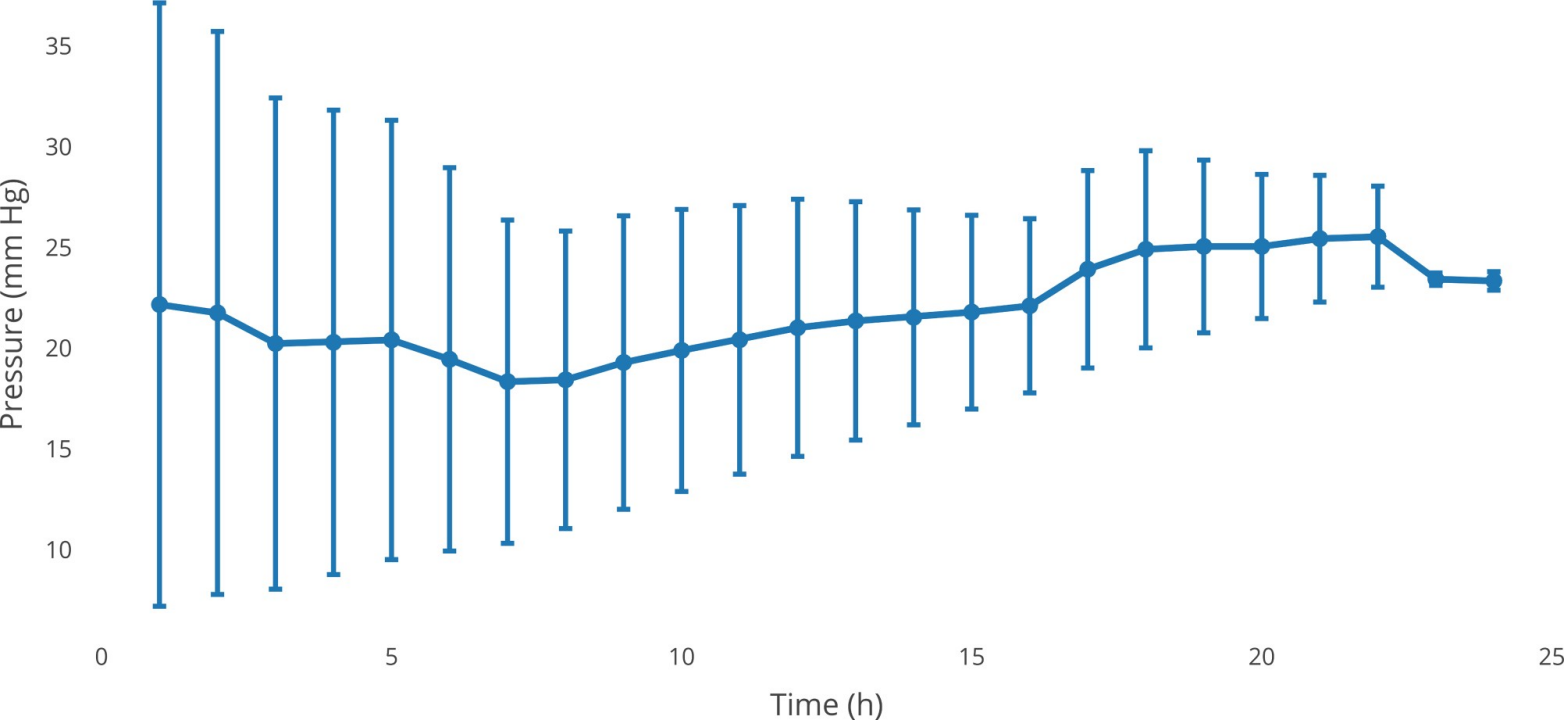
Effects of VBS on the IOP in the porcine vitrectomy model. Average of series (n = 5). Representative standard deviations for each measurement are given.

For evaluation of the effects of the generated artificial VBS on IOP, an explanted pig eye model was used. After a core vitrectomy including careful removal of the peripheral vitreous base and a fluid-air exchange, VBS was injected via a 20-gauge Weber-needle. The procedure was performed by two experienced vitreoretinal surgeons. The injection was performed manually without any problems. Continuous IOP measurements (Fig 2) revealed a 5% decrease 12 h after VBS instillation followed by an increase of 10% after 24 h. This effect was, however, not statistically significant (p>0.05).

**Fig 3:**
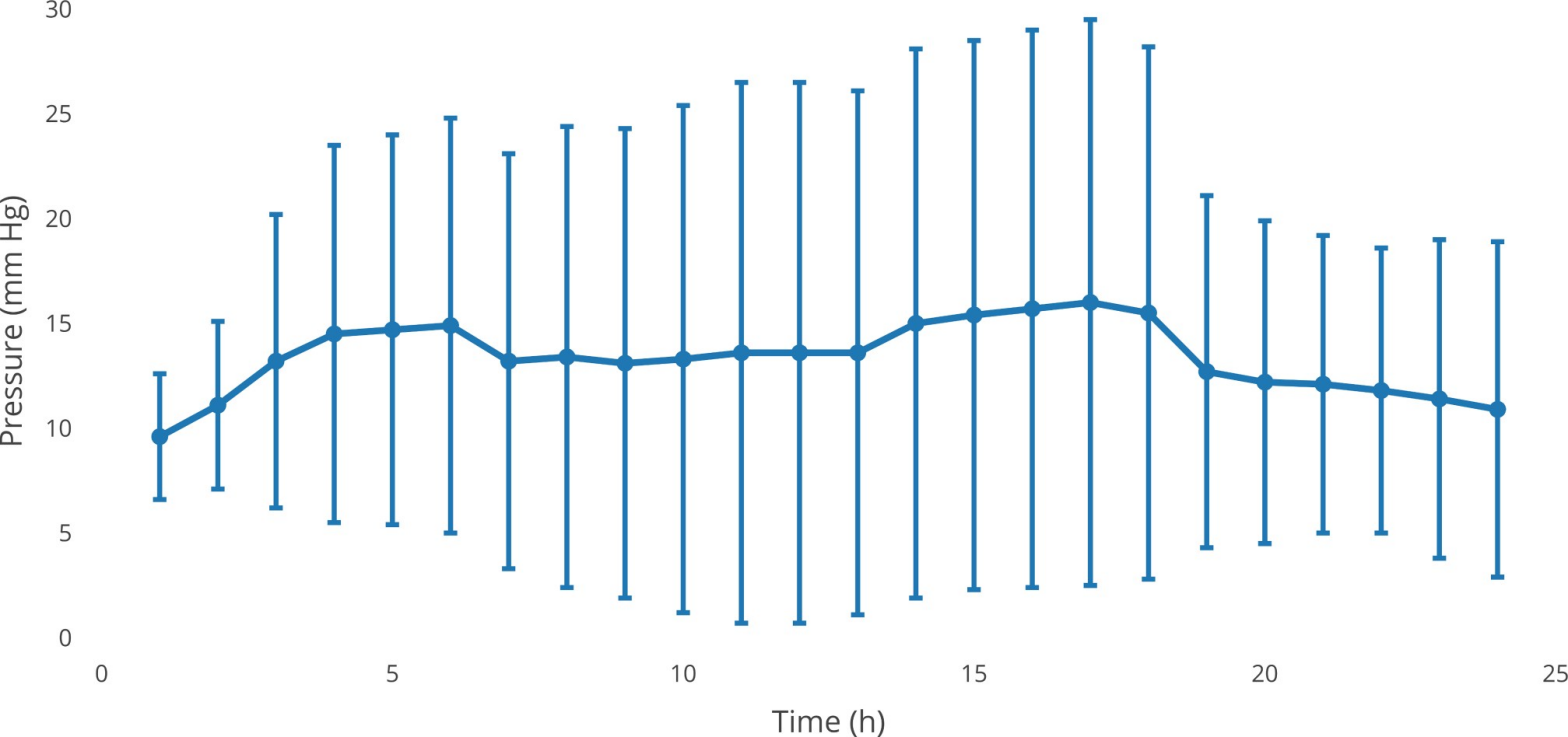
Effects of BSS serving as control on the IOP in the porcine vitrectomy model. Average of series (n = 5). Representative standard deviations for each measurement are given.

After injection of only BSS, an increase of 42% after 12 h was noted, and a 13% increase was observed after 24 h (p>0.05, Fig 3).

### Evaluation of PV after 24 h regarding biocompatibility

The Bax/Bcl-2-expression level ratio as a marker for apoptosis did not show any significant difference between BSS-treated eyes set as 1.0 or eyes treated with VBS after 24 h of exposure (0.88; p > 0.05, n = 5). No signs of astrocyte or Müller cell activation were observed via qRT-PCR of GFAP expression in hydrogel-treated vs BSS-treated eyes, which were set as 1.0 (1.39; p > 0.05, n = 5). No significant differences in β-3–tubulin protein expression in hydrogel-treated versus BSS-treated were seen on western blotting (b—3 –tubulin ration of 0.94, p > 0.05, n = 4), and no significant decrease of PGP9.5 mRNA expression (1.13, p > 0.05, n = 5) was observed, indicating that there had not been any loss of retinal ganglion cells.

### Evaluation of VBS effects on retinal function

**Fig 4:**
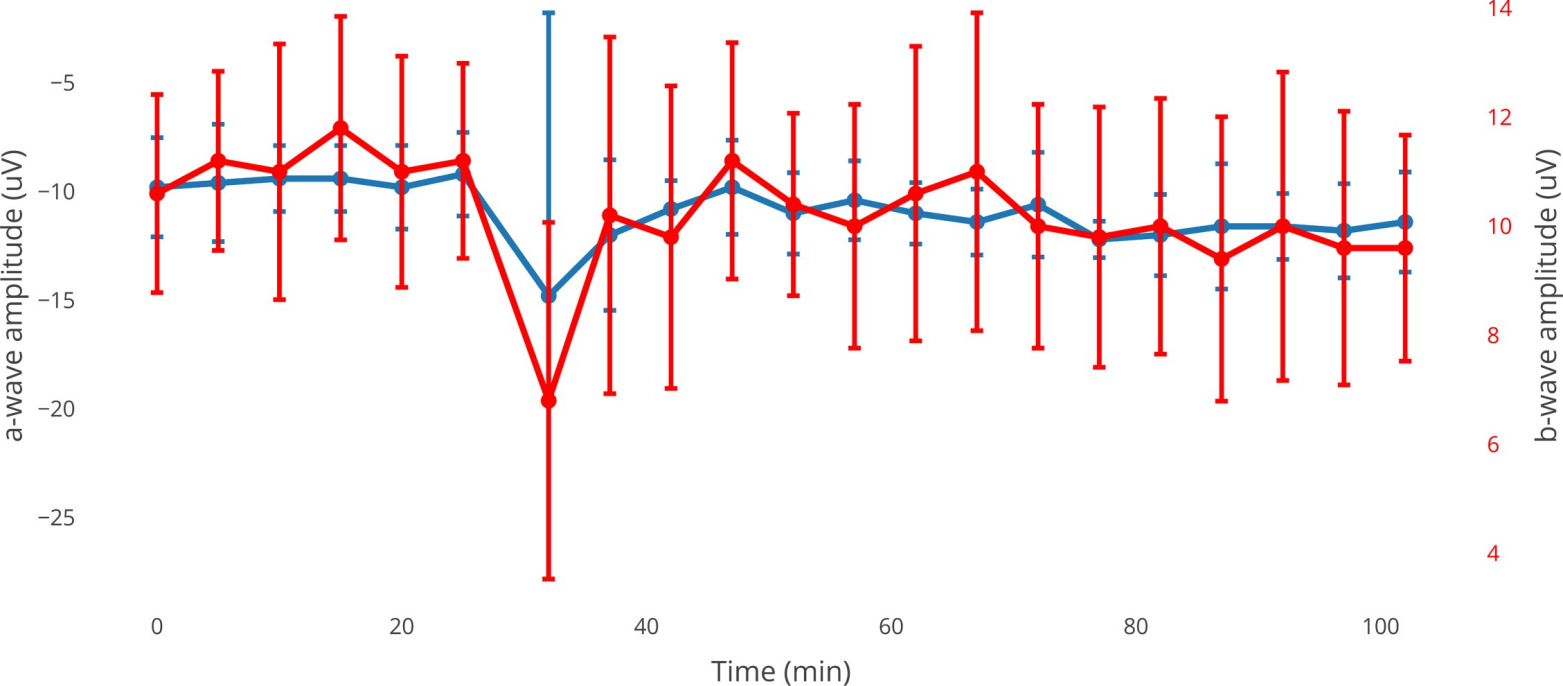
Effects of VBS on the a- and b-wave amplitude of the ERG taken from isolated superperfused bovine retinae. Average of series (n = 5). The blue line represents the a-wave amplitudes, the red line represents the b-wave amplitudes. Representative standard deviations for each drug series are given.

In addition to the effects of VBS implantation on IOP, compatibility of the hydrogel with retinal function was investigated. For this purpose, an ex vivo bovine retina model was used, and the influence of applied VBS on electrophysiological parameters was studied. ERGs (a- and b-waves) taken from the superfused retina before application were compared to measurements shortly after VBS application and at the end of a 70-min washout period. Five minutes after exposing the retina for 120 seconds to VBS, a 9% reduction of the b-wave amplitude was observed, which was, however, not statistically significant (p>0.05, Fig 4). Similarly, a non-significant reduction of the amplitude by 14.2% was registered at the end of the washout period (p>0.05, Fig 4). The measurements before application served as a control, as usual.

During the testing of the photoreceptor potential measured by the a-wave amplitude, an increase of 30.4% directly after VBS application was noted, which was again not statistically significant. At the end of the observation period, the value was increased by 23.9%, which also did not reach statistical significance (p>0.05, Fig 4). Mean values and standard deviations (SD) for b-wave and a-wave amplitudes are given in Table 2.

**Table 2:** Photoreceptor potential after vitreous body substitute application.

Time [min]	b-wave amplitude [μV]	SD	a-wave amplitude [μV]	SD
0	10.6	1.816590212	-9.8	2.28035085
5	11.2	1.643167673	-9.6	2.701851217
10	11	2.34520788	-9.4	1.516575089
15	11.8	2.049390153	-9.4	1.516575089
20	11	2.121320344	-9.8	1.923538406
25	11.2	1.788854382	-9.2	1.923538406
32	6.8	3.271085447	-14.8	13.02689526
37	10.2	3.271085447	-12	3.464101615
42	9.8	2.774887385	-10.8	1.303840481
47	11.2	2.167948339	-9.8	2.167948339
52	10.4	1.673320053	-11	1.870828693
57	10	2.236067977	-10.4	1.816590212
62	10.6	2.701851217	-11	1.414213562
67	11	2.915475947	-11.4	1.516575089
72	10	2.236067977	-10.6	2.408318916
77	9.8	2.387467277	-12.2	0.836660027
82	10	2.34520788	-12	1.870828693
87	9.4	2.607680962	-11.6	2.880972058
92	10	2.828427125	-11.6	1.516575089
97	9.6	2.50998008	-11.8	2.167948339
102	9.6	2.073644135	-11.4	2.302172887

## Discussion

Facing the demand for distinct vitreous substitutes with better biocompatibility, less vision disturbance and defined stability [17], a newly developed hydrogel generated from thiolated HA was evaluated for its biophysical characteristics in two ex vivo eye models. Physicochemical characterization of the naturally cross-linked gel revealed a refractive index almost identical to the human vitreous. The transparent, hydrophilic material was shown to be highly elastic but still easily injectable, with the needle diameters usually being applied during vitrectomy. The shear moduli are comparable to other novel vitreous substitutes [18,19]. The goal of this study was not only to evaluate the potential of VBS for in vivo testing; it also shows a simple and cost-effective way to eliminate prototypes with unfavourable short term characteristics before going into the expensive and highly regulated animal-testing phase. The choice of two ex vivo models (porcine/bovine) might be an impacting factor, since they were used for different tests, and it would be advisable to choose only one species in order to increase standardization. However, while the porcine model anatomically resembles the human eye more closely, it is not possible to obtain postmortem ERGs (due to the use of electroshocks) from the porcine model.

The moderate, statistically non-significant increase in IOP determined from the porcine anterior chamber model corresponds well with data from in vitro swelling assays, indicating a maximum weight increase (wet versus dry) of the tested hydrogel by 50% after 48 h. This swelling is comparable to existing hydrogels and lower than existing tamponades such as silicone oils [11,18–20].

The used ex vivo pig eye model is a variation of the well described anterior chamber model established by Johnson et al. [17] and modified by Shaarawy et al. [21] for testing the influence of glaucoma surgery on IOP. We recently evaluated the potential of this porcine model for IOP monitoring during vitrectomies using conventional vitreous tamponades and found it suitable [22]. One might argue that not removing the lens might change IOP fluctuations significantly, but fortunately, we showed in an earlier study that this was not the case [11]. Using this model, in the present study, we could show that there was no significant increase in intraocular pressure within 24 h after implantation of VBS. This result is comparable to the findings of existing in vivo studies [20]. With respect to IOP, the first 24 h after vitreoretinal surgery are the most critical [23]; therefore, this time interval was chosen. In addition, most of the hydrogel swelling occurs during the first 24 h. Thus, the ex vivo porcine eye model allows for screening for potentially adverse effects of swelling hydrogels while reducing the need for animal experiments. However, investigation of long-term IOP effects will still require in vivo analysis as the hydrogel slowly dissolves, and degradation products may adversely affect IOP or retinal function.

The isolated bovine retina, which shows significant analogies to the human retina, is a sensitive and well-evaluated tool for short term pharmacological testing and, in this respect, has shown comparable results to human isolated organ cultures [16,24]. A possible toxic effect of a tested substance on retinal neurons can be detected as a reduced electrical signal. Additionally, the site of action can be differentiated by isolating the photoreceptor potential using aspartate. Moreover, compared to animal experiments, this ex vivo model shows a high level of standardization [25] Accordingly, it seemed highly appropriate to test the retinal biocompatibility of the newly developed VBS in this model.

The obtained results demonstrated that neither a-wave nor b-wave amplitudes were affected significantly by short-term application of the hydrogel. The high standard deviation noticed shortly after exposition of the retina with the vitreous substitute is caused by the experimental setup and has been discussed earlier [26, 27]. Thus, the important reference for monitoring changes is the amplitude recorded at the end of the washout period which, in the present experiments, did not show any significant alterations.

Although ERG recordings from superfused retinae are very valuable for a functional analysis on cellular level, longer exposition periods than 12 h cannot be tested under these highly standardized conditions. Moreover, the exposure time of the hydrogel in the present experiments was limited to 120 seconds due to its highly viscous nature, which did not allow for continuous superperfusion of the system. Since ex vivo retinal viability is strongly dependent on a sufficient supply of oxygen and nutrients, longer exposure times were not feasible. Moreover, the model of the superfused retina primarily provides information about the integrity of the outer retina; the effect of VBS on the inner retina, e.g., ganglion cells, cannot be sufficiently evaluated by this means. Thus, biocompatibility testing of the new VBS prototype beyond the 2-min exposure period will depend on an in vivo model. To overcome this shortcoming in our setup, qRTPCR and western blot analysis were performed over an exposure period of 24 h using PV, indicating good biocompatibility. It is arguable that the clinical exposure time is considerably longer; therefore, in vivo biocompatibility experiments are recommended. The presented ex vivo data, however, underline the potential of this HA-based VBS and PV for vitreoretinal surgery.

In summary, the manufactured thiolated HA-based hydrogel showed highly favourable biophysical characteristics in the explored ex vivo models, strongly suggesting its further application as a vitreous substitute in vivo.

## References

[1] Kleinberg TT, Tzekov RT, Stein L, Ravi N, Kaushal S. Vitreous substitutes: a comprehensive review. Survey of ophthalmology. [Review]. 2011 Jul-Aug;56(4):300–23. 10.1016/j.survophthal.2010.09.001 21601902

[2] Spitzer MS, Januschowski K. [Aging and age-related changes of the vitreous body]. Der Ophthalmologe: Zeitschrift der Deutschen Ophthalmologischen Gesellschaft. 2015 7;112(7):552, 4–8. 26002378 10.1007/s00347-015-0031-9

[3] Le Goff MM, Bishop PN. Adult vitreous structure and postnatal changes. Eye. [Review]. 2008 10;22(10):1214–22. 10.1038/eye.2008.21 18309340

[4] Suri S, Banerjee R. In vitro evaluation of in situ gels as short term vitreous substitutes. Journal of biomedical materials research Part A. 2006 12 1;79(3):650–64. 10.1002/jbm.a.30917 16826595

[5] Su X, Tan MJ, Li Z, Wong M, Rajamani L, Lingam G, et al Recent Progress in Using Biomaterials as Vitreous Substitutes. Biomacromolecules. 2015 10 12;16(10):3093–102. 10.1021/acs.biomac.5b01091 26366887

[6] Gao QY, Fu Y, Hui YN. Vitreous substitutes: challenges and directions. International journal of ophthalmology. 2015;8(3):437–40. 10.3980/j.issn.2222-3959.2015.03.01 26085987 PMC4458642

[7] Nakagawa M, Tanaka M, Miyata T. Evaluation of collagen gel and hyaluronic acid as vitreous substitutes. Ophthalmic research. [Comparative Study]. 1997;29(6):409–20. 10.1159/000268042 9380343

[8] Pruett RC, Schepens CL, Swann DA. Hyaluronic acid vitreous substitute. A six-year clinical evaluation. Archives of ophthalmology. [Research Support, U.S. Gov't, P.H.S.]. 1979 12;97(12):2325–30. 518384 10.1001/archopht.1979.01020020541006

[9] Schramm C, Spitzer MS, Henke-Fahle S, Steinmetz G, Januschowski K, Heiduschka P, et al The cross-linked biopolymer hyaluronic acid as an artificial vitreous substitute. Investigative ophthalmology & visual science. [Comparative Study]. 2012 2;53(2):613–21. 22199245 10.1167/iovs.11-7322

[10] Spitzer MS, Sat M, Schramm C, Schnichels S, Schultheiss M, Yoeruek E, et al Biocompatibility and antifibrotic effect of UV-cross-linked hyaluronate as a release-system for tranilast after trabeculectomy in a rabbit model—a pilot study. Current eye research. 2012 6;37(6):463–70. 10.3109/02713683.2012.658593 22577763

[11] Schnichels S, Schneider N, Hohenadl C, Hurst J, Schatz A, Januschowski K, et al Efficacy of two different thiol-modified crosslinked hyaluronate formulations as vitreous replacement compared to silicone oil in a model of retinal detachment. PLoS One. 2017; 12(3): e0172895 10.1371/journal.pone.0172895 28248989 PMC5332068

[12] Johnson DH, Tschumper RC. The effect of organ culture on human trabecular meshwork. Experimental eye research. [Research Support, Non-U.S. Gov't Research Support, U.S. Gov't, P.H.S.]. 1989 7;49(1):113–27. 2759186 10.1016/0014-4835(89)90080-8

[13] Bachmann B, Birke M, Kook D, Eichhorn M, Lutjen-Drecoll E. Ultrastructural and biochemical evaluation of the porcine anterior chamber perfusion model. Investigative ophthalmology & visual science. [Research Support, Non-U.S. Gov't]. 2006 5;47(5):2011–20. 16639010 10.1167/iovs.05-1393

[14] Ebner M, Mariacher S, Hurst J, Szurman P, Schnichels S, Spitzer MS, et al Characterization of a Standardized Ex-vivo Porcine Model to Assess Short Term Intraocular Pressure Changes and Trabecular Meshwork Vitality After Pars Plana Vitrectomy with Different Silicone Oil and BSS Tamponades. Curr Eye Res. 2017 8;42(8):1130–1135. 10.1080/02713683.2017.1297461 Epub 2017 Apr 25. 28441060

[15] Januschowski K, Muller S, Krupp C, Spitzer MS, Hurst J, Schultheiss M, et al Glutamate and hypoxia as a stress model for the isolated perfused vertebrate retina. Journal of visualized experiments: JoVE. [Video-Audio Media]. 2015(97). 10.3791/52270 PMC4401365 25868118

[16] Januschowski K, Zhour A, Lee A, Maddani R, Mueller S, Spitzer MS, et al Testing the biocompatibility of a glutathione-containing intra-ocular irrigation solution by using an isolated perfused bovine retina organ culture model—an alternative to animal testing. Alternatives to laboratory animals: ATLA. [Evaluation Studies]. 2012 3;40(1):23–32. 22558975 10.1177/026119291204000107

[17] Mariacher S, Szurman P. [Artificial vitreous body: Strategies for vitreous body substitutes]. Der Ophthalmologe: Zeitschrift der Deutschen Ophthalmologischen Gesellschaft. 2015 7;112(7):572–9. 26077344 10.1007/s00347-015-0057-z

[18] Santhanam S, Liang J, Struckhoff J, Hamilton PD, Ravi N. Biomimetic Hydrogel with Tunable Mechanical Properties for Vitreous Substitutes. Acta Biomater. 2016 10 1; 43: 327–337. 10.1016/j.actbio.2016.07.051 27481290 PMC5787031

[19] Swindle KE, Hamilton PD, Ravi N. In situ formation of hydrogels as vitreous substitutes: Viscoelastic comparison to porcine vitreous. J Biomed Mater Res A. 2008 12 1;87(3):656–65. 10.1002/jbm.a.31769 18189301

[20] Schramm C, Spitzer M, Henke-Fahle S, Steinmetz G, Januschowski K, Heiduschka P, et al The Cross-linked Biopolymer Hyaluronic Acid as an Artificial Vitreous Substitute. Investigative Ophthalmology & Visual Science February 2012, Vol.53, 613–621. 10.1167/iovs.11-7322 22199245

[21] Shaarawy T, Wu R, Mermoud A, Flammer J, Haefliger IO. Influence of non-penetrating glaucoma surgery on aqueous outflow facility in isolated porcine eyes. The British journal of ophthalmology. 2004 7;88(7):950–2. 10.1136/bjo.2003.035535 15205245 PMC1772209

[22] Januschowski K, Mariacher S, Ebner M, Schnichels S, Hurst J, Szurman P, et al An ex-vivo model for the short term IOP development for vitreous tamponades. Invest Ophthalmol Vis Sci 2015 p. 211.

[23] Wong R, Gupta B, Williamson TH, Laidlaw DA. Day 1 postoperative intraocular pressure spike in vitreoretinal surgery (VDOP1). Acta ophthalmologica. 2011 6;89(4):365–8. 10.1111/j.1755-3768.2009.01703.x 19860785

[24] Mukhopadhyay A, Gupta A, Mukherjee S, Chaudhuri K, Ray K. Did myocilin evolve from two different primordial proteins? Molecular vision. [Comparative Study Research Support, Non-U.S. Gov't]. 2002 7 22;8:271–9. 12142865

[25] Richter SH, Garner JP, Wurbel H. Environmental standardization: cure or cause of poor reproducibility in animal experiments? Nature methods. [Research Support, Non-U.S. Gov't]. 2009 4;6(4):257–61. 10.1038/nmeth.1312 19333241

[26] Luke M, Weiergraber M, Brand C, Siapich SA, Banat M, Hescheler J, et al The isolated perfused bovine retina—a sensitive tool for pharmacological research on retinal function. Brain research Brain research protocols. [Research Support, Non-U.S. Gov't]. 2005 12;16(1–3):27–36. 10.1016/j.brainresprot.2005.09.001 16275053

[27] Luke M, Januschowski K, Beutel J, Luke C, Grisanti S, Peters S, et al Electrophysiological effects of Brilliant Blue G in the model of the isolated perfused vertebrate retina. Graefe's archive for clinical and experimental ophthalmology = Albrecht von Graefes Archiv fur klinische und experimentelle Ophthalmologie. [Research Support, Non-U.S. Gov't]. 2008 6;246(6):817–22. 10.1007/s00417-007-0761-8 18197412

